# Supplementary figures and images for: Effects of Larval Density on Gene Regulation in Caenorhabditis elegans During Routine L1 Synchronization
Source: G3 (Bethesda). 2018 Mar 30;8(5):1787–93. doi: 10.1534/g3.118.200056 (PMC5940168; doi:10.1534/g3.118.200056)

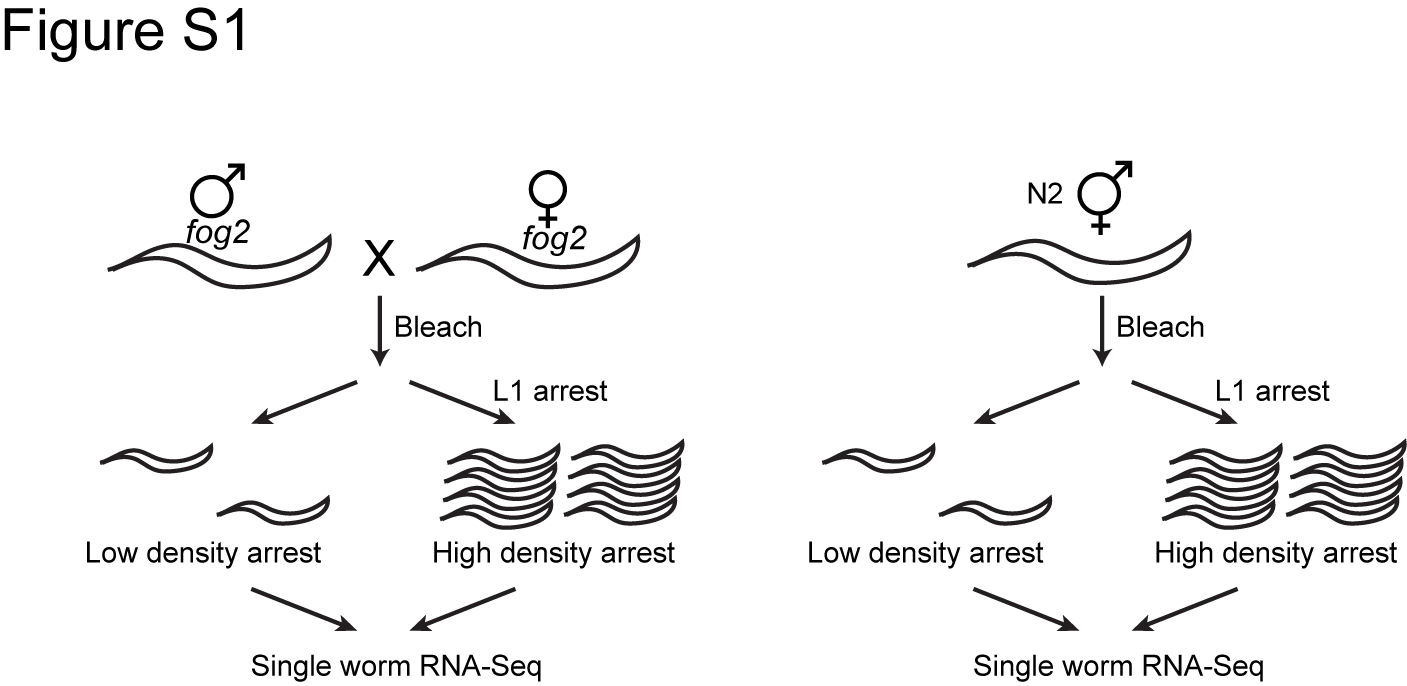

Supplement: Supplementary file 1 [file 1787FigureS1.tif]

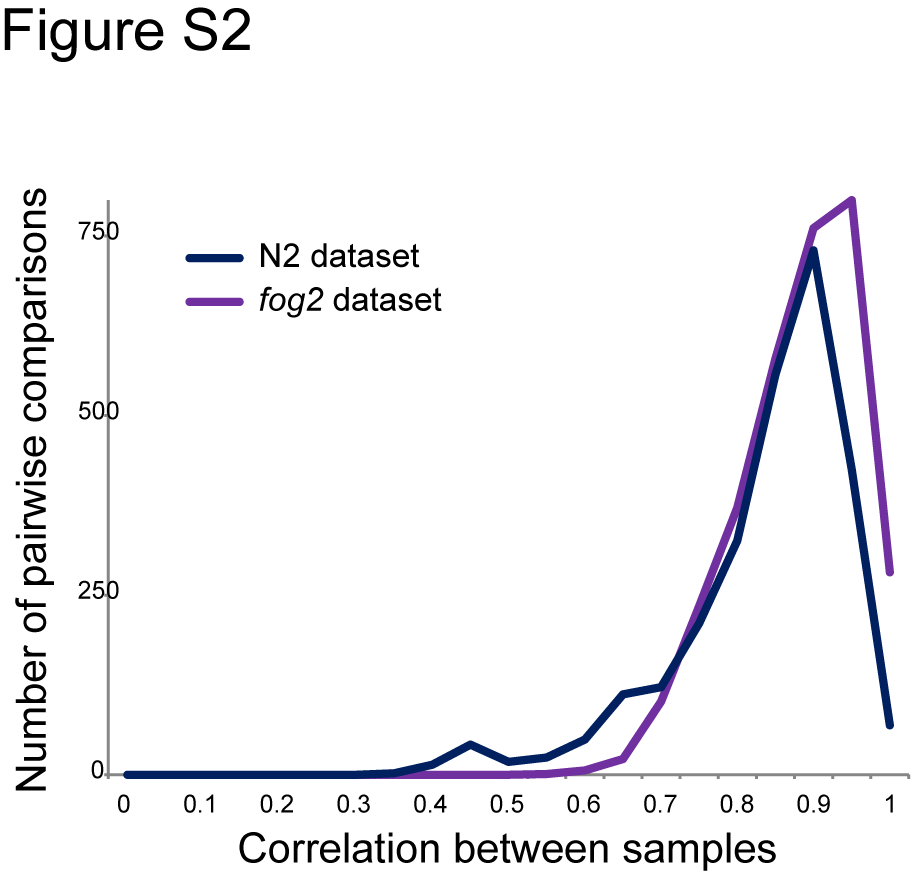

Supplement: Supplementary file 2 [file 1787FigureS2.tif]

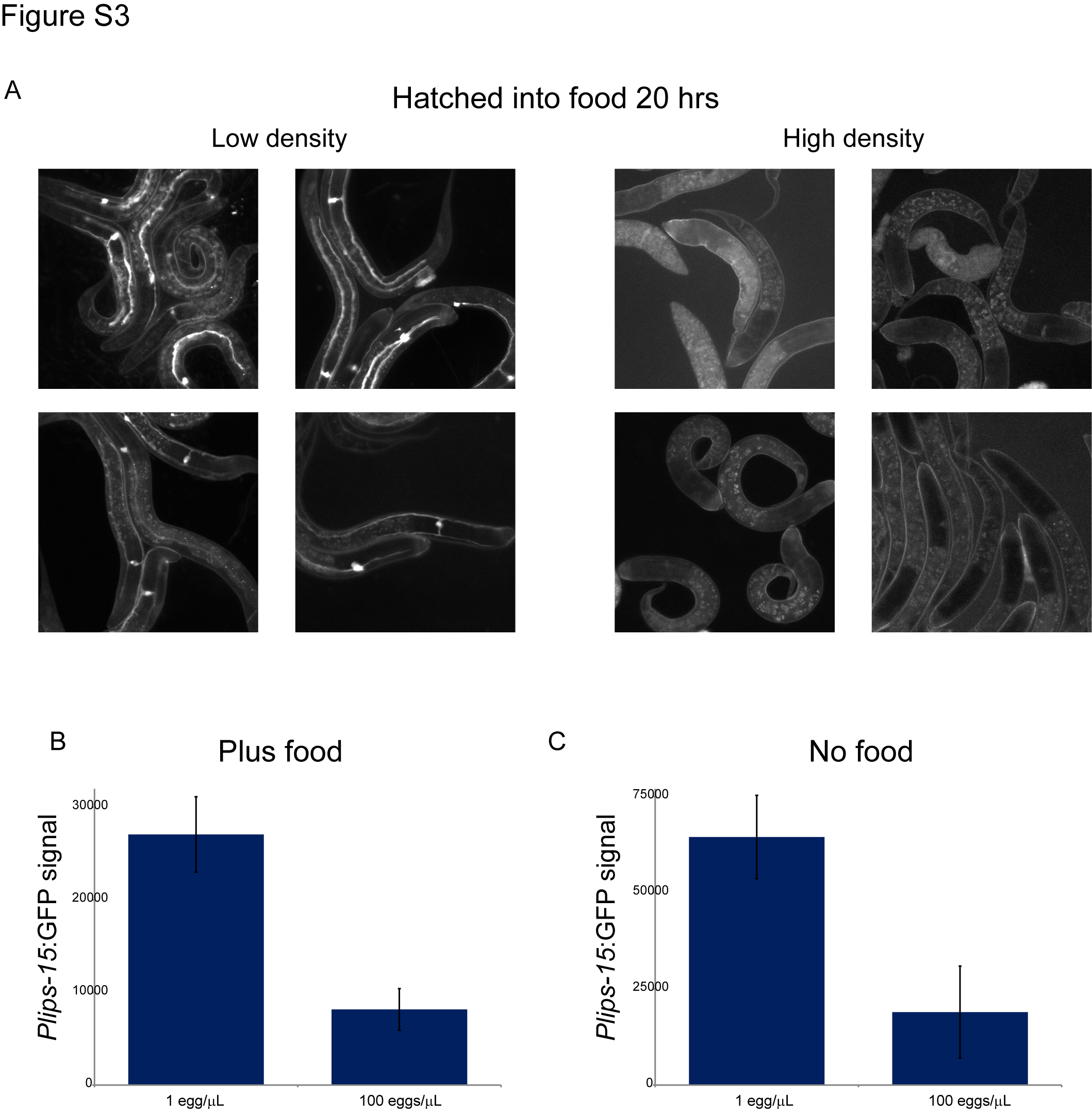

Supplement: Supplementary file 3 [file 1787FigureS3.tif]
